# Enhancing Deep-Learning Classification for Remote Motor Imagery Rehabilitation Using Multi-Subject Transfer Learning in IoT Environment

**DOI:** 10.3390/s24248127

**Published:** 2024-12-19

**Authors:** Joharah Khabti, Saad AlAhmadi, Adel Soudani

**Affiliations:** 1 College of Computer and Information Sciences (CCIS), King Saud University, Riyadh 11543, Saudi Arabia; salahmadi@ksu.edu.sa (S.A.); asoudani@ksu.edu.sa (A.S.); 2 King Salman Center for Disability Research, Riyadh 11614, Saudi Arabia

**Keywords:** brain–computer interface (BCI), motor imagery (MI), electroencephalogram (EEG), deep learning (DL), transfer learning (TL), internet of things (IoT), edge computing

## Abstract

One of the most promising applications for electroencephalogram (EEG)-based brain–computer interfaces (BCIs) is motor rehabilitation through motor imagery (MI) tasks. However, current MI training requires physical attendance, while remote MI training can be applied anywhere, facilitating flexible rehabilitation. Providing remote MI training raises challenges to ensuring an accurate recognition of MI tasks by healthcare providers, in addition to managing computation and communication costs. The MI tasks are recognized through EEG signal processing and classification, which can drain sensor energy due to the complexity of the data and the presence of redundant information, often influenced by subject-dependent factors. To address these challenges, we propose in this paper a multi-subject transfer-learning approach for an efficient MI training framework in remote rehabilitation within an IoT environment. For efficient implementation, we propose an IoT architecture that includes cloud/edge computing as a solution to enhance the system’s efficiency and reduce the use of network resources. Furthermore, deep-learning classification with and without channel selection is applied in the cloud, while multi-subject transfer-learning classification is utilized at the edge node. Various transfer-learning strategies, including different epochs, freezing layers, and data divisions, were employed to improve accuracy and efficiency. To validate this framework, we used the BCI IV 2a dataset, focusing on subjects 7, 8, and 9 as targets. The results demonstrated that our approach significantly enhanced the average accuracy in both multi-subject and single-subject transfer-learning classification. In three-subject transfer-learning classification, the FCNNA model achieved up to 79.77% accuracy without channel selection and 76.90% with channel selection. For two-subject and single-subject transfer learning, the application of transfer learning improved the average accuracy by up to 6.55% and 12.19%, respectively, compared to classification without transfer learning. This framework offers a promising solution for remote MI rehabilitation, providing both accurate task recognition and efficient resource usage.

## 1. Introduction

Motor imagery is one of the main applications used in brain–computer interfaces. Through motor imagery, a person can mentally simulate specific movements without physically executing them. This concept could aid in the control of assistive devices for individuals with disabilities, as well as in the rehabilitation of injured individuals to improve muscle strength or to manage pain. EEG signals, which are commonly used to measure motor imagery activity in the brain, play a crucial role in BCIs, clinical diagnostics, and neuroscience research. These signals are characterized by their high temporal resolution, allowing for real-time monitoring of brain activity. EEG signals have demonstrated their efficacy across various fields, such as stroke rehabilitation [1,2], wheelchair control [3], robot control [4], and other assistive technologies. However, EEG signals are non-stationary, meaning their statistical properties can change over time, which further complicates the classification process [5]. Additionally, the challenge is heightened when dealing with limited data for a person, making accurate analysis and classification of EEG signals for motor imagery tasks even more difficult.

Consider developing an online motor imagery rehabilitation system that incrementally gathers patient data over time. The main difficulty in designing such systems is accurately identifying the motor imagery tasks performed by the patient, particularly in the initial stages of rehabilitation when patient-specific data are limited. To address this, we adopted a pre-trained model to provide preliminary predictions about motor imagery tasks, especially during the initial sessions. This model can then be fine-tuned with the patient’s specific data to improve its accuracy—a process known as transfer learning [6]. Transfer learning is a crucial technique in deep learning, especially due to the limited availability of EEG data and the extensive datasets typically required by neural network models [7]. It is also valuable in scenarios where the initial data for classification is lacking. However, the non-stationary nature of EEG signals poses significant challenges to the process of transfer learning. EEG signals may vary considerably between trials or even within the same trial for the same subject [5]. Nevertheless, subjects tend to display consistent EEG patterns when mentally simulating the same movement [8].

Transfer learning (TL) is a well-studied technique for adapting models trained on one subject to another related subject. Various strategies have been employed to improve accuracy for the target subject, including methods such as freezing specific layers [9], subject-to-subject semantic-style transfer network (SSSTN) [10], and approaches based on Riemannian geometry [6]. While TL is typically applied to a single target subject [6,7,8,10], the concept can be extended to multiple target subjects within the same region, offering opportunities to address broader variability and improve performance. Research indicates that cultural backgrounds can influence brain activity patterns. For instance, researchers in [11] have shown differences in EEG patterns between individuals from different cultural backgrounds during cognitive tasks. The authors of [12] provided a meta-analysis that explores how cultural differences affect human brain activity, which includes discussions on motor imagery. Moreover, the researchers in [13] emphasized that BCI applications should account for cultural differences, considering how linguistic and cultural factors affect BCI performance and usage. Consequently, we propose to use distinct models retrained in different regions to account for cultural differences. This approach represents a key distinction between our work and previous studies, where multiple subjects were used as the target in transfer learning.

The architecture of the online rehabilitation system requires a multi-layered structure. At the cloud level, pre-trained models are generated, and distinct models are distributed to each region for retraining at the patient level. Data collection in this system is performed by IoT devices and sensors, including EEG sensors, which gather real-time patient information. This data is then transmitted to the cloud for processing. However, transmitting large volumes of patient data to the cloud necessitates efficient management and control [14,15]. Additionally, IoT devices face inherent limitations, such as restricted computational power, memory, bandwidth, and energy resources, which complicate the processing of such extensive data [16]. These challenges call for solutions that distribute computational tasks across multiple layers. The authors in [17] declared a need to build a new network level in order to decrease the load on the cloud and perform the complex computational process. Researchers have proposed architectures that integrate cloud, fog, and edge computing to address these challenges and support IoT applications [18,19,20,21]. The cloud serves as a centralized hub for model training, while fog and edge layers process data closer to the source, reducing delays and decreasing the amount of data transmitted. As highlighted in [17], both fog and edge computing facilitate data processing between IoT devices and the cloud, offering faster analysis and localized decision-making. Fog nodes specialize in aggregating information from geographically dispersed devices, making them suitable for extensive systems but often difficult to manage. In contrast, edge computing is more compatible with our proposed architecture, as it supports real-time processing of EEG data and delivers immediate feedback to patients. In this context, we propose an efficient IoT framework for remote training based on edge/cloud computing as three network layers to cooperate and balance data processing. The classification process of EEG signals is applied at the edge level to overcome the communication cost and to provide the patient with a quick response. Moreover, the edge level can enhance MI accuracy by implementing subject-specific training.

Recognizing the patient’s motor imagery (MI) tasks remains a critical challenge due to the complexity of EEG signals. Various techniques were proposed in the literature to address this challenge, including feature extraction methods such as common spatial patterns (CSP) combined with machine-learning models like support vector machines (SVMs) and linear discriminant analysis (LDA) [22,23,24]. Convolutional neural networks (CNNs) have also been explored as solutions, with examples including a dual-branch CNN with a self-attention mechanism [25] and a multi-branch one-dimensional CNN with residual blocks [26]. Moreover, one effective approach using fusion CNN with attention blocks (FCNNA) provided a well-performing classification model, as demonstrated in our previous work [27]. In this study, we build upon that work by incorporating transfer learning. Additionally, EEG signals often contain redundant channels that can negatively affect accuracy and efficiency. To mitigate this issue, our approach minimizes the number of EEG channels used, thereby optimizing both accuracy and efficiency. Moreover, various transfer strategies, such as adjusting epochs, freezing layers, and employing different data division techniques, are applied to enhance both efficiency and accuracy. Both the classification and channel selection methods are derived from this robust framework [27]. Consequently, our system’s effectiveness in addressing these challenges through the use of different classification models, channel selection algorithms, and transfer-learning strategies highlights its potential for improving the accuracy and efficiency of EEG-based MI task recognition.

The main contributions of this paper are as follows:

1. To develop an online rehabilitation system with an edge-computing layer to provide an efficient IoT framework for remote training. This layer reduces communication and computational costs on central servers;
2. To introduce a multi-subject transfer-learning process, where the target subjects include more than one subject for retraining the same model alternately. This approach enhances MI accuracy, optimizes memory, and considers common cultural differences while maintaining only one model per region;
3. To improve efficiency and accuracy by applying a freezing strategy and selecting a unified set of optimal channels for all subjects, which optimizes memory usage and computation time;
4. To provide a comprehensive comparison for cross-subject classification for each model and transfer-learning strategy used, exploring their respective advantages and disadvantages.

The rest of the paper is organized as follows. Section 2 reviews the solutions required for edge computing within an IoT framework and briefly describes the current transfer-learning classification techniques employed for EEG signals. Section 3 provides a detailed explanation of the methods and architectures proposed in this study. In Section 4, the data and the models of the system are analyzed. The experimental results and discussion are presented in Section 5. Finally, the paper is concluded in Section 6.

## 2. Related Works

### 2.1. IoT and Edge Computing

IoT devices face challenges while collecting, processing, and transmitting massive amounts of data due to their limited resources. This section will demonstrate the possible solutions for distributing the IoT procedures to be handled by different computing layers.

The authors in [14] illustrated that cloud computing could overcome IoT limitations and provide the extra resources needed. However, this solution suffers from traffic delays and communication costs due to the long distance between the cloud and the sensors. Furthermore, Paul et al. clarified in [15] that relying on cloud computing carries the risk of data center failure, as millions of IoT devices communicate with the central cloud. Therefore, edge and fog computing are alternative technologies that are employed to provide services to IoT devices with faster response and greater quality, as declared in [28].

The authors of [18,19] proposed a fog-computing strategy to provide privacy in IoT environments. Fog nodes connect sensors to the cloud and vice versa. Moreover, each group communicates with other groups in order to distribute the workload and aggregate the data. On the other hand, the authors of [20] implemented an edge-computing architecture to build an air pollution monitoring system efficiently. Edge nodes collect data from sensors and respond with an immediate notification after quick preprocessing. Later, edge devices transmit the data-processing results to the cloud. Moreover, Alonso et al., in [21], proposed a platform for smart farming environments where farm sensors send data to the edge layer in order to manipulate the data analysis and then later send the output to the cloud. The edge is used to reduce the volume of data transferred to the cloud and minimize the use of cloud resources.

The authors of [17] highlighted the differences between fog and edge layers. Edge and fog computing are similar in that they operate as a bridge between IoT devices and cloud computing, allowing faster data analysis. Nevertheless, fog nodes play a role in data aggregation via connecting groups of fog devices. They can gather information from a variety of sensors spread across a large geographic area. But, the fog layer is built from multiple geographically distributed devices, which requires significant effort to design a suitable architecture and to discover a method for sharing end-user resources [17].

In the realm of edge computing, numerous studies have leveraged edge architecture to enhance smart health systems and classification tasks. For example, the authors of [29] proposed a multi-access edge-computing (MEC)-based architecture for e-health applications, utilizing hospitals as edge nodes to facilitate rapid detection and response. This architecture was designed for the real-time detection and monitoring of various data and sensors associated with different diseases, such as cardiac disorder detection. Moreover, smart healthcare applications utilizing edge computing were proposed by [30] for remote monitoring and seizure. They implemented an edge layer using a Samsung smartphone, achieving 98.3% seizure classification accuracy, a 60% extension of battery life, and a 90% reduction in transmission delay. Furthermore, the authors of [31] suggested using a virtual machine (VMware) as an edge node to train EEG signals for exploring IoT applications, with a focus on time efficiency. They employed an extended particle swarm optimization (PSO)-based neural network for motor imagery (MI) classification, achieving an accuracy of 98.9%.

Our work distinguishes itself from previous studies by being applied to an online e-rehabilitation system, where edge computing is leveraged to accelerate the process and retrain the model according to specific regions. At the edge level, EEG data is collected from individuals within the same geographical area to facilitate localized training and classification. Primary health centers (PHCs) in each district serve as the edge nodes in this system.

### 2.2. Transfer Learning

A review of the related research on brain–computer interfaces (BCIs) is presented in this section, with a particular emphasis on the implementation of transfer learning (TL) in systems based on motor imagery (MI). The researchers in [7] evaluated transfer learning using two scenarios: within-subject and cross-subject. In the within-subject scenario, transfer learning was applied individually to each subject across different sessions, with the first session being used for training, and a pre-trained classifier was applied to subsequent sessions. In the cross-subject scenario, the classifier was trained on data from all subjects except the target subject and, then, adapted for the target subject’s sessions. They used both online and offline learning methods to retrain the model, achieving up to a 3% improvement in decoding accuracy across three models. The authors of [9] proposed an adaptive transfer-learning technique that fine-tunes a pre-trained model using a portion of the target subject’s data before validation. This technique involves freezing different layers of the model’s weights to retain useful features from the source domain while adapting them to the target domain. They used the DeepConvNet [32] architecture to classify two tasks for one target subject based on a model pre-trained on data from other subjects.

Specifically, in the BCI IV 2a dataset, numerous papers have applied several transfer-learning techniques to enhance the accuracy of four-class classification using various methods. According to [10], researchers developed a subject-to-subject semantic-style transfer network (SSSTN) for multiclass MI classification. They employed continuous wavelet transform (CWT) to convert EEG data into images. The SSSTN performs style transfer between subjects, generating class-discriminative data while considering input data semantics. The generator aligns each participant’s data individually as a target with the style of a single source data for subject-to-subject style transfer. The source subject classifier accurately classifies the transformed target data, achieving an accuracy of 80.66%. As indicated in [33], the authors introduced the Siamese deep domain adaptation (SDDA) framework, validated with the EEGNet and ConvNet models. They utilized transfer learning to improve classification performance, incorporating maximum mean discrepancy (MMD) loss to align the embedding features between the source and target domains from the same participant. The accuracy achieved by the SDDA with ConvNet surpasses that of EEGNet, reaching 82.01% compared to 79.43% for the four-class classification task. The authors of [34] utilized two domain adaptation techniques to conduct transfer learning through deep-learning classification in order to distinguish between four tasks. The first adaptation technique involves choosing similar data from the source domain, while the second adaptation technique is aimed at reducing the differences between the source and target domains. In applying these adaptation techniques, FBCNet was utilized to extract features from the data, followed by a dual classifier based on deep learning. For the BCI IV 2a dataset, the first session of each subject was used as the source and the second session as the target. As a result, the authors achieved an accuracy rate of 81%. According to [8], the authors applied multi-direction transfer learning (MDTL) to categorize four tasks for cross-subject strategy. Their method involves multiple rounds of model training and retraining to generate a variety of new models from random source subjects. The target subject data is then evaluated through some of these newly generated models to identify the most suitable model for each subject. A new model is chosen for evaluation when the target subject data is included in the source domain. The authors of [35] improved a two-class cross-subject classification method for MMFT, extending its application to multi-class classification problems, as demonstrated by the examples involving three and four classes. Furthermore, they employed an optimal combination of sources to produce multiple classifiers for transfer mapping. These classifiers align the distribution means between the source and target domains for prediction, with the results determined by the most frequent label. According to [36], the authors proposed a CNN network that combines time convolution and EEGNet to extract and merge features from the source and target subjects. CORAL and classification loss functions are used to optimize the classification accuracy. In their experiment, they used a single source subject for each target subject and calculated the average results, focusing on subjects 1, 3, 7, 8, and 9. For domain adaptation challenges, the authors of [37] introduced a machine-learning framework that integrates graph neural networks (GNNs) with transfer-learning techniques. Subjects from certain datasets were used as the source, while subjects from other datasets served as the target. Transfer learning was applied by fine-tuning the pretrained model using a subset of the subjects from the target datasets.

Several papers, such as [6,38,39,40,41], utilize Riemannian geometry and alignment techniques to address the differences between subjects in transfer learning for EEG classification. The authors of [38] enhanced the classification performance of the PLVQ-LEML Riemannian classifier by applying two transfer-learning methods, PA-LEM and RPA-LEM. These methods, based on the log-Euclidean metric, are used to reduce the distribution differences between the source data and the target data. In their cross-subject classification, data from one subject were used as the training set, while data from another subject were used as the test set. According to the authors of [39], they proposed a double-stage transfer-learning (DSTL) algorithm that applies transfer learning in both the preprocessing and feature extraction stages. The approach involves aligning EEG trials using Euclidean alignment (EA). It then reweights the source domain trials based on the distance between their covariance matrices and the mean covariance matrix of the target domain. The method uses two feature extraction stages. It reduces domain differences with a transfer component analysis (TCA) after extracting the features with common spatial patterns (CSP). Finally, an LDA classifier is used for classification. The authors of [6] proposed a multi-source fusion transfer-learning (MFTL) algorithm, which performs cross-subject classification for four classes by utilizing a portion of the target data for training through transfer learning. Due to the insufficient training data available for the target subject, the authors selected a group of source subjects that were expected to enhance performance. They employed the minimum distance to Riemannian mean (MDRM) method to optimally choose the subjects that provided the highest accuracy by evaluating the classification results. Additionally, they used Riemannian geometry alignment (RGA), a transfer-learning algorithm for Riemannian manifolds that normalizes the covariance matrices of different subjects to reduce the differences between the source and target subjects. According to the BCI IV 2a dataset, the optimal number of source subjects is two, and this approach resulted in an average accuracy of 62.81%. By applying MDLT through the EEGNet [42] classifier, the accuracy results reached 75%. By incorporating information from a resting state, the authors of [40] proposed a transfer-learning (TL) framework designed to enhance the performance of minimum distance to Riemannian mean (MDRM) classifiers. They worked in the Riemannian space and aligned covariance matrices from different sessions and subjects to provide a common reference. This method, termed Riemannian Alignment (RA) MDRM, demonstrated improved accuracy in the classification of four classes of cross-subject and cross-session motor imagery tasks. However, because classification is performed in the Riemannian space, where geodesic computation is more complex, time-consuming, and unstable, ref. [41] addressed these challenges by proposing an alternative approach. According to [41], Euclidean alignment (EA) simplifies the process of transforming and aligning EEG signals in Euclidean space for feature extraction, signal processing, and classification. The proposed EA-CSP-LDA algorithm outperformed RA-MDRM in several aspects, achieving an average cross-subject accuracy of 73.53% on the BCI IV 2a dataset, compared to 70.91% for RA-MDRM.

Based on the existing literature, various techniques have been applied to enhance performance using transfer learning. These include freezing layers, using different learning rates, splitting data into different portions, and developing methods to transfer features from multiple sources. However, our research differs from previous studies by including multi-subjects as a target and employing a channel selection algorithm to maximize efficiency. Additionally, we improve the model’s performance and efficiency in distinguishing between four classes by freezing certain layers and fine-tuning the remaining ones with different learning rates.

## 3. Materials and Methods

This section illustrates the architecture of the proposed online rehabilitation system and provides descriptions of the techniques used in transfer-learning classification at the edge level.

### 3.1. Online Rehabilitation (OR) System Architecture

**Figure 1:**
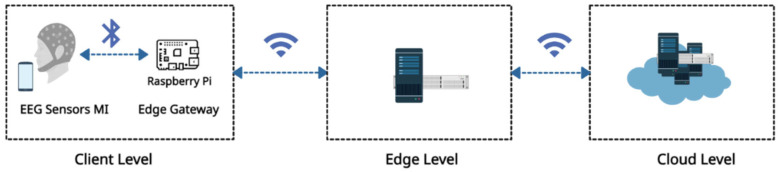
Data flow and devices of the online rehabilitation (OR) system.

The architecture of the proposed system is depicted in Figure 1, illustrating the main components of the online rehabilitation network and its layers. The system comprises three primary layers: cloud, edge, and client. The edge layer is included to enhance efficiency, providing quick responses while maintaining the specific characteristics of each region. The figure demonstrates the direction of data flow and the protocols used for transactions. According to Figure 1 a brief description of each layer is as follows:

Client Level (Sensors)

At the Client Level, EEG sensors for MI are attached to the user’s headset to capture brain signals. These sensors are connected via Bluetooth to an edge gateway, which includes a Raspberry Pi device responsible for initial data processing and transmission. This level primarily focuses on collecting raw data from the sensors and performing preliminary processing such as extracting windows of 4.5 s from each trial and dropping the selected channels. The processed data are then transmitted to the edge node for further analysis.

Furthermore, the rehabilitation instructions for each session, such as ‘imagine moving your right hand’, are generated by the main hospital and stored in the cloud. These instructions are then sent to the care provider’s server at the edge node. From there, they are delivered to the patient’s phone prior to the rehabilitation session. The patient uses these instructions to perform specific motor imagery tasks during their rehabilitation exercises.

Edge Level

The edge level consists of an edge node (regional server) that acts as an intermediary between the client devices and the cloud infrastructure. Communication between the edge node, the client (via the edge gateway), and the cloud is facilitated through Wi-Fi. The edge node provides additional processing power and storage to handle data efficiently. Processing data locally at the edge node ensures rapid response times and preserves the unique characteristics of each region. It reduces communication overhead and offloads computational burden from the cloud. In the proposed framework, a regional care provider’s server acts as the edge node for retraining the model with local patient data. The updated model parameters are saved at the edge node and used exclusively for that region. This decentralized approach ensures that each region has a model fine-tuned to its specific patient population, enhancing the accuracy and effectiveness of predictions during rehabilitation training while reducing the need for extensive data transmission to the main hospital. For a detailed discussion on the retrained model and transfer-learning strategies, refer to Section 3.2.

Cloud Level

The cloud represents the servers of the primary rehabilitation service provider (i.e., the main hospital). The main service provider is responsible for providing the trained model and the common channels to the edge nodes in each region. The trained model assists in predicting tasks for new patient data during rehabilitation training. Meanwhile, the channels are selected to minimize the amount of information transmitted through the network, based on the optimal channel selection methodology outlined in [27]. This approach enhances efficiency while maintaining a high level of accuracy. Also, the cloud servers receive performance feedback on the patients’ training sessions from each edge node.

To ensure reliable WiFi transmission between the cloud, edge, and client levels, mechanisms such as automatic repeat request (ARQ) and acknowledgment signals could be employed. These strategies help detect and recover from potential packet loss or interference, ensuring the integrity of the transmitted data [43].

### 3.2. Transfer-Learning Framework and Strategies

In the edge node, transfer learning is deployed to retrain the model, adapting it to the patients’ data in the edge region. Following the principles of transfer learning, we built and compared the performances of four models developed for this purpose. Two of these models replicated the structure used in our earlier study [27]: one without channel selection and one with channel selection employing a cross-subject strategy. The other two models are similar to the previous ones, differing only in their kernel size values. This adjustment reduces processing time, effectively addressing the challenges posed by large data volumes. The process of choosing the models and the main comparison between the performances of the models are shown in Section 4.3. Here is a list of the models used:

1. FCNNA Model without channel selection [27];
2. FCNNA Model with channel selection [27];
3. LFCNN Model without channel selection;
4. LFCNN Model with channel selection.

The previously listed models are structured with two layers of convolutional blocks, each followed by CBAM attention mechanisms. Each convolutional layer comprises two distinct blocks, with one employing both frequency and spatial filters, and the other utilizing a separable convolutional block. Furthermore, a genetic algorithm is implemented to reduce the number of channels. By selecting one set of optimal channels, the same channels are used for all subjects during training [27]. The overall architecture of the FCNNA and LFCNN models remains consistent, with the primary variation being the kernel sizes, as detailed in Section 4.3, Table 2.

It is important to note that all of the aforementioned methods have been utilized to generate multiple pre-trained models. In our approach, which employs a cross-subject strategy, each model is trained on a subset of source subjects and subsequently tested on a different source subject. These pre-trained models are subsequently deployed in a transfer-learning process to adapt to the data of the target subjects. Based on the accuracy and efficiency results from the transfer-learning experiments conducted in this study, the optimal retrained model will be selected for preparation in the cloud and then deployed to the edge nodes.

The following sections focus on describing and clarifying the methodologies used in the proposed transfer-learning process. We begin by providing a comprehensive overview of the general framework for EEG classification in a subject-specific context, incorporating both pre-trained and retrained models. Additionally, we will outline the strategies employed to update the model at the edge node, including the transfer-learning online mode, the session-based data division strategy, and the freezing strategy. These strategies are essential for enhancing the model’s adaptability and performance, ensuring that it can effectively learn from new data in real-time while maintaining stability and accuracy.

#### 3.2.1. General Framework of Subject-Specific Classification

**Figure 2:**
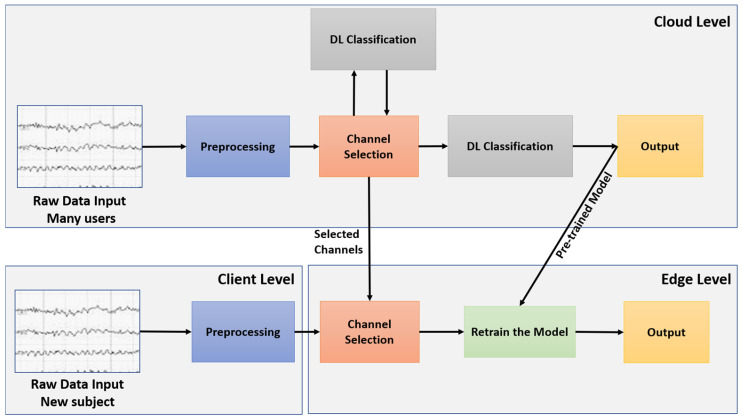
Subject-specific classification framework.

The framework of the selected models consists of three main processes: channel selection, preprocessing, and classification. Figure 2 illustrates the deployment of transfer learning. It shows the primary steps of EEG classification across the client, edge, and cloud levels. Raw EEG data from source subjects are received and preprocessed at the cloud level, while data from the target subjects are received and preprocessed at the client level. At the client level, the data (from patient rehabilitation sessions) are preprocessed before being sent to the edge level. Both the cloud and edge levels process data through a series of steps, including channel selection and deep-learning classification. At the cloud level, these processes are applied to the source subjects to identify the optimal channels using a channel selection algorithm and to develop a pre-trained subject-specific classification model through deep learning. This pre-trained model, along with the optimal channels, is then sent to the edge level. At the edge level, the selected channels and the preprocessed target data are used to retrain the pre-trained model through transfer learning, adapting it to the specific characteristics of the target subjects.

#### 3.2.2. Transfer-Learning Online Mode

**Figure 3:**
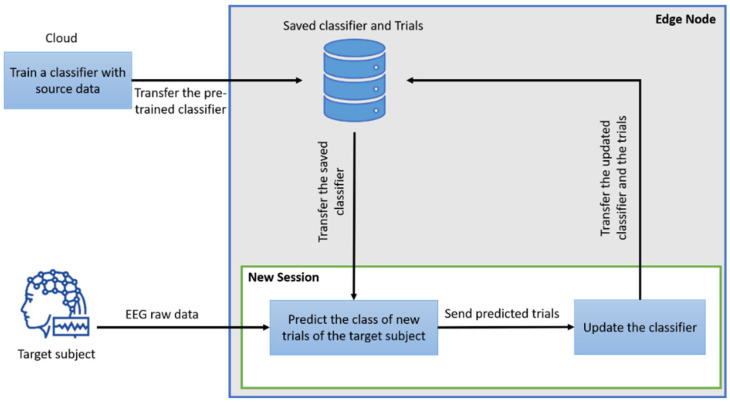
Online mode process.

In this paper, we use the online mode [7] in transfer learning to enhance our model, facilitating real-time data processing and online rehabilitation. This approach involves incrementally updating the model with new data as it becomes available, rather than retraining it with a static dataset. This continuous learning process allows us to adapt our model to new patterns and variations of data in real-time, maintaining robustness and accuracy. Figure 3 illustrates the implementation of the online mode. Initially, a classifier is trained using a cross-subject strategy, where one of the source subjects is tested at a time in the cloud. Subsequently, the classifier is transferred to the edge node for use in new sessions with the target subjects. Throughout the process, a list of predicted trials, initially empty, is maintained along with the saved classifier. At the beginning of each session with the target subject, predictions are made using the saved classifier, which first predicts the class of several trials. Taking advantage of the new data, the classifier is then updated based on these new predicted trials. This process is repeated for each new session. The saved classifier predicts the new trials and is subsequently updated according to the newly available data. At the end of each session, the new predicted trials are saved. Moreover, if the classifier demonstrates improved performance at the end of the session, it will also be saved on the edge node server. In general, the online mode reduces computational overhead and facilitates real-time adaptation, making it an optimal choice for our study, especially in dynamic and data-rich environments. The system was not specifically examined in an online setting, but it closely mimics the dynamics of an online learning environment.

#### 3.2.3. Session-Based Data Division Strategy

In this section, we describe the methodology used to divide the data into multiple sessions, each trained separately to facilitate the transfer-learning process. Our approach involves training different target subjects at different times using the same model. By selecting the data size for each session, we effectively determine the data size for each training period. The model is updated and retrained with each new session, incorporating new data to progressively improve its performance. This approach ensures that the model receives diverse and comprehensive training inputs from different subjects on the edge node, which helps manage the training process efficiently and enhances the model’s ability to generalize across different data segments.

**Figure 4:**
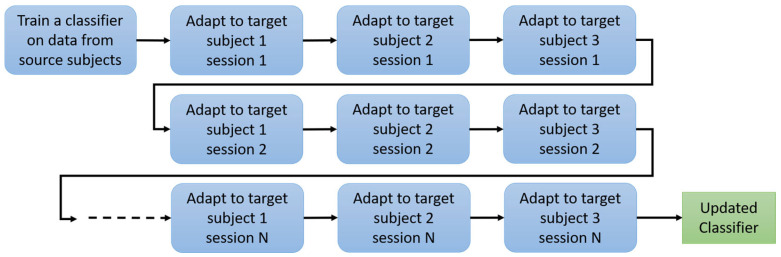
Data division strategy among different targets and sessions. At each stage, the classifier is retrained and sent from one point to another until the process is completed.

Figure 4 illustrates how each session, containing data from each subject, is trained at alternating times through transfer learning with the same model, which is continuously updated and retrained. In this figure, we consider three subjects on the edge node with N sessions, where N is the division size of the rehabilitation training. The division strategy is crucial for optimizing transfer learning and achieving high accuracy in subject-specific EEG classification. In the experiment, we will demonstrate two values of N and how they affect performance.

#### 3.2.4. Freezing Strategy in Transfer Learning

**Figure 5:**
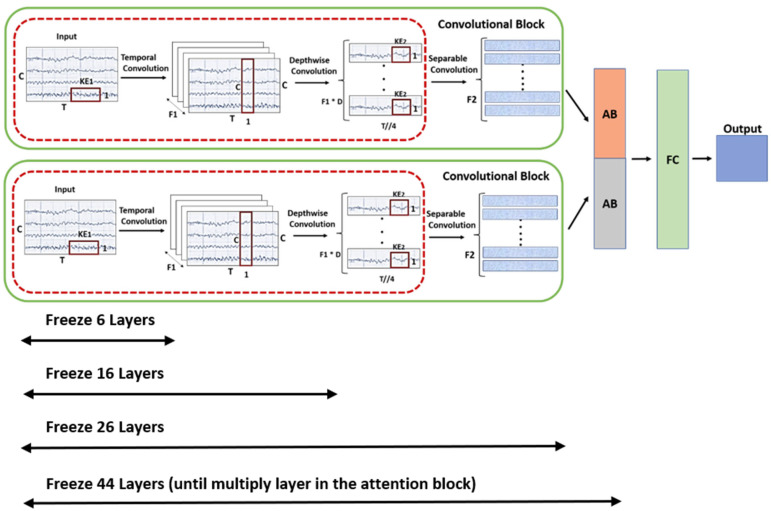
Levels of frozen layers, where AB = attention block and FC = fully connected of FCNNA model described in [27].

In the proposed methodology, we use a freezing strategy as part of the transfer-learning approach to fine-tune the model efficiently. We start by freezing the parameters of some initial layers of the pre-trained model and retraining the parameters of the remaining layers to adapt the model to new data or new subjects. To optimize this fine-tuning process, we also adjust the model’s configuration parameters by applying different levels of freezing over various numbers of epochs and reducing the learning rate as needed. The levels of freezing applied in this work are shown in Figure 5. We use four different levels of freezing, each designed to preserve specific parameter data according to the FCNNA model used in [27]. In the beginning, we applied six layers of freezing where only time-domain filters were preserved to maintain the initial feature extraction. By freezing these layers, we ensure that the fundamental temporal patterns learned from the original dataset are retained, providing a stable foundation for further processing. At the second level, the parameters of the initial sixteen layers are frozen. These layers typically include time-domain filters and spatial filters from the pre-trained model. By freezing these layers, we retain the model’s ability to extract fundamental temporal and spatial features from the input EEG signals, ensuring that the basic structure and essential features learned from the original dataset are preserved. By freezing the initial layers, including the separable convolutions (up to layer 26), we ensure the preservation of the parameters responsible for extracting higher-order temporal and spatial features. In the final stage, 44 layers are frozen, which include parts of the attention block. By freezing these layers, we maintain the model’s ability to focus on significant features learned from the original dataset, ensuring that important aspects of the data are emphasized while adapting to new tasks. Applying different levels of freezing allows us to make meaningful comparisons and assess the impact of each freezing level on model performance. This approach helps in understanding how retaining specific learned features affects the model’s ability to adapt to new datasets or subjects. Additionally, it helps identify the most efficient freezing strategy for achieving optimal performance with minimal computational cost.

By applying transfer learning with different model configurations, including varying levels of freezing, epochs, and learning rates, we can thoroughly compare their effects on performance. By adjusting the learning rate across different ranges, we can identify the optimal rate that preserves the model’s learned weights and prevents disruptive updates to the optimized parameters. Additionally, by carefully selecting the number of epochs, we can control the extent of training, allowing the model to converge appropriately without overfitting. This balance between learning rate and epochs is crucial for maintaining the integrity of the pre-trained features while adapting the model to new data. Similarly, we will apply an unfreezing strategy, adjusting the same parameters (epochs and learning rates) to provide a comprehensive comparison. This approach allows us to evaluate the impact of different training strategies on the model’s ability to adapt and generalize to new data.

### 3.3. Stages and Key Contributions in the Online Rehabilitation(OR) Models

**Figure 6:**
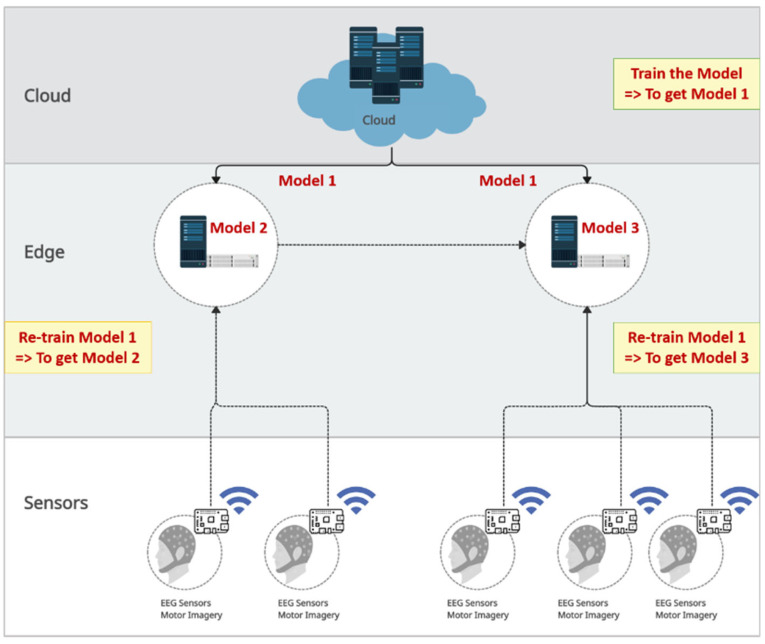
Distribution of the models in each layer of the online rehabilitation (OR) scheme.

As illustrated in Figure 6, the models are generated within the online rehabilitation architecture. Model 1 is generated in the cloud as the main pre-trained model and then distributed to all of the edge nodes. Each edge node receives new data specific to the targets of its respective region and subsequently retrains Model 1 to generate updated models. Consequently, each node generates a different model, with Model 2 produced by the first edge node and Model 3 produced by the second edge node. Later, as new data from their respective regions are received, each edge node uses its own model (Model 2 or Model 3) for prediction and further retraining.

Table 1 presents three key points of our approach regarding the model generation process using user data. (1) The data of the target subject are excluded from training the pre-trained model (Model 1). (2) The model is retrained. (3) The transfer learning is applied to multiple subjects. The table highlights the effectiveness of our approach by emphasizing these key aspects and showcasing the novelty of our method compared to previous studies. Unlike most studies, our work does not include any data from the target subjects in the pre-trained model. Some studies inconsistently incorporate target data into pre-trained models, but only in works [37,41,43]. The target data are fully excluded during pre-training. Also, our model is retrained using continuous data from the target subjects, a practice rarely employed, except in study [7], or four-class experiments and study [37] for two-class experiments. The main difference between our study and the prior research contribution is that we applied transfer learning to multiple target subjects, whereas previous studies were limited to a single target subject.

## 4. System Analysis and Model Evaluation

### 4.1. Dataset of BCI Competition IV 2a [45]

**Figure 7:**
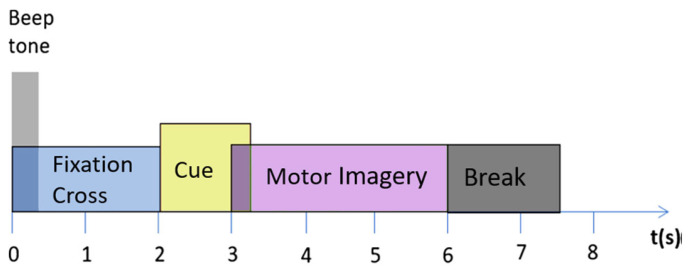
Timing scheme of the BCI IV 2a dataset.

The BCI IV 2a dataset was recorded with 22 electrodes to collect EEG data from nine subjects who were positioned according to the International 10–20 system. The data include recordings of four different motor imagery tasks: imagining the movement of the left hand (class 1), right hand (class 2), both feet (class 3), and tongue (class 4). Each of the nine subjects participated in two separate recording sessions held on different days. In each session, 288 trials were conducted, which were divided into six runs of 48 trials each covering all four classes. The EEG signals were sampled at 250 Hz and filtered with a bandpass filter between 0.5 Hz and 100 Hz. As depicted in Figure 7, each trial lasts approximately 7 s, including a four-second imagination period following the cue onset, and concludes with a break.

In our study, we divided the subjects into two groups: source subjects and target subjects. The first six subjects are designated as source subjects, while the remaining subjects are designated as target subjects. This grouping was based on findings from our previous work [27] and related studies [36,38], which showed that subjects 1, 3, 7, 8, and 9 achieved the best classification performance. Specifically, our earlier experiments demonstrated that the classifiers trained on subjects 1 and 3 performed better in cross-subject classification, both with and without channel selection, across all nine subjects. Based on these findings, subjects 1 and 3 are recommended as source subjects, while subjects 7, 8, and 9 are well-suited for testing. These results provided a logical basis for the chosen subject grouping.

According to the data sessions, the two sessions were grouped and then divided into four or six divisions, each treated as a stand-alone rehabilitation session. For subjects 7, 8, and 9, the combination of the two sessions will produce 548 trials as the maximum. After the division, we will get either 140 or 90 trials for each rehabilitation session where each trial lasts for 7 s. As a result, each rehabilitation session will last either 17 or 11 min.

### 4.2. IoT and Edge-Computing Evaluation

To validate the feasibility of the proposed system, we simulated the performance of the client and edge layers using the datasets used in this paper. At the client level, we evaluated the suitability of Raspberry Pi devices and Bluetooth 5.0 for handling EEG data transmission and preprocessing. Calculations of memory usage and transmission times demonstrate that Raspberry Pi 4B has sufficient capacity to manage data from various channel configurations (22, 13, 12, or 9 channels) sampled at 250 Hz. For example, transmitting data from 22 channels requires approximately 41.12 MB of memory for one rehabilitation session and about 1.23 s per trial for Bluetooth transmission, which is well within the device’s capabilities. This evaluation confirms that the proposed architecture can efficiently handle the expected data flow, ensuring real-time processing and transmission within the system. Detailed calculations and simulations are provided in Appendix A for further reference.

In general, we prove that Raspberry Pi 4B, with its ample memory capacity and Bluetooth 5.0 support, is well-suited to handle the expected EEG data volume and transmission requirements for the online rehabilitation system. By transmitting each trial separately, we ensure efficient data processing and real-time transmission within the system, making it a viable solution for the intended application. This approach effectively manages the data flow, reduces potential delays, and maintains system performance.

Meanwhile, at the edge node servers, information from multiple patients will be received. It is crucial to reduce the data volume as much as possible to handle processing and data management in real-time efficiently. Reducing the number of channels helps minimize transmission consumption. For more information on how we increase efficiency using different strategies, starting with selecting a model that optimizes time, accuracy, and computational efficiency, please refer to Section 4.3.

### 4.3. Evaluate the Efficiency Between Models Used

As previously discussed, we chose to apply transfer learning through two primary models: the FCNNA model and the LFCNN model. In this section, we detail the process of selecting these two models from a generated set of three. Initially, we used the FCNNA model as described in [27] and developed two additional models (XFCNN and LFCNN) by tuning certain hyperparameters to enhance efficiency. Consequently, we had three main models for comparison, from which we selected two. The key differences between the models are the hyperparameters listed in Table 2. This table shows the variations in F1 and kernel-width 1 (KE1) values for the first and second layers. KE1 in the convolutional layers specifies the number of consecutive temporal samples analyzed for each electrode, directly influencing the feature extraction process from EEG signals. These variations lead to the distinct outcomes presented in Table 3. It is important to note that, for all of the models, the following values were maintained: D = 2 in the first layer, D = 1 in the second layer, and KE2 = 16.

Table 3 provides a comparative analysis of the three models, illustrating the efficiency of each based on key metrics, such as the number of trainable parameters, multiply-accumulate operations (MAC), within-subject classification accuracy, and training time. The number of trainable parameters indicates the network’s size and impacts memory usage, while the MAC metric represents the network’s arithmetic complexity, directly influencing latency and power consumption [46]. It is also important to note that floating-point operations (FLOP) are another metric used to estimate power and latency. However, based on previous studies, FLOP is typically either equivalent to or twice the value of MAC [46]. The MAC is calculated using the following formula [47]:

$$Conv2D=K_{1}\times K_{2}\times C_{in}\times C_{out}\times H_{out}\times W_{out}$$

$$SeparableConv2D=\left(K_{1}\times K_{2}+C_{out}\right)*C_{in}\times H_{out}\times W_{out}$$

$$DepthWiseConv2D=K_{1}\times K_{2}\times C_{in}\times D\times H_{out}\times W_{out}$$

According to the data presented in Table 3, The FCNNA model, with 362.32 KB of trained parameters and 184 million MACs, achieved the highest within-subject classification accuracy at 83.78% but required the longest training time of 4 h and 34 min. The XFCNN model, which uses 50.37 KB of trained parameters and 50 million MACs, attained an accuracy of 82.11% with a reduced training time of 2 h. The LFCNN model, the most efficient with 32.55 KB of trained parameters and 35 million MACs, resulted in an accuracy of 81.09% and a training time of 1 h and 56 min. Based on these results, we decided to select the FCNNA model for its highest accuracy and the LFCNN model for its efficiency. This selection allows us to balance performance and computational efficiency in our experiments.

To illustrate the effectiveness of our selection of the FCNNA and LFCNN models, we conducted cross-subject classification across all subjects, particularly since our transfer-learning approach focuses on cross-subject classification. The mean results analysis revealed that the FCNNA model achieved an accuracy of 68.87%, while the LFCNN model attained a higher accuracy of 70.20%. In light of this, it appears that the LFCNN model would be a good alternative to complement the existing FCNNA model.

## 5. Experiments and Discussions

In this study, we built the FCNNA and LFCNN models within the same environment described in [27]. The models were implemented in Python using TensorFlow (version 2.15.0) and deployed on Google Colab, equipped with a T4 GPU and 15.0 GB of GPU RAM. To achieve reliable outcomes, we conducted three runs for each transfer-learning experiment, which involved varying model architectures, division sizes, learning rates, numbers of epochs, and levels of freezing, as detailed in this section. During training, we employed a callback function to save the model weights at the point of highest average accuracy across the target subjects’ trials, thus optimizing the use of computational resources.

We utilized an online strategy using two division levels on subjects seven, eight, and nine. We divided each subject’s data, which consisted of approximately 600 samples, into groups for training and prediction. Using the six-division strategy, the data were divided into six groups (0–90, 90–180, 180–270, 270–360, 360–450, 450–600), which were used for prediction and retraining. In the four-division strategy, the data were split into four groups (0–140, 140–280, 280–420, 420–600) for prediction and retraining. During retraining, the first group of samples was run using five epochs to gradually introduce the data into the classifier. Afterward, the runs were conducted with one of the following epoch settings: (30, 50, 100, 200).

### 5.1. Preparation of Pre-Trained Models

Initially, to begin working with transfer learning, we prepared the pre-trained classifiers. As noted in Section 3.2, we developed four models:

1. FCNNA Model without channel selection [27];
2. FCNNA Model with channel selection [27];
3. LFCNN Model without channel selection;
4. LFCNN Model with channel selection.

A cross-subject classification across all source subjects for each model was adopted. In this approach, one subject was used for testing while the remaining subjects were utilized for training. This technique enables the model to train on and learn the majority of the features from the source subject data. Consequently, each of the four listed models generated six classifiers, resulting in a total of 24 classifiers. Specifically for the models using channel selection, we applied a genetic algorithm following the methodology described in [27]. The genetic algorithm was employed to identify the optimal channels for testing each subject from the source data. As a result, different combinations of channels produced varying accuracies, and the channels with the highest accuracy were selected as the optimal channels for testing that subject. The selected channels were determined after applying the algorithm two to three times. Subsequently, cross-subject classification was conducted to test each subject using the selected optimal channels for that subject. It was performed six times, either with or without channel selection, and the classifier with the best accuracy was chosen. The final results are presented in Table 4, which shows the accuracy results for each classifier when testing a single subject across the six source subjects, both with and without channel selection. Additionally, the optimal channels identified using the genetic algorithm are listed.

To select the best classifier for transfer learning from each set of six classifiers, we applied two criteria. First, we chose the classifier with the highest accuracy. Second, we selected the classifier that showed the greatest improvement in accuracy after applying channel selection. Based on the results presented in Table 4, it is obvious that testing on subject three yields the highest accuracy across all four models. Additionally, subject 1 shows the highest increase after applying channel selection (CS), with a 5.06% improvement in FCNNA and a 2.34% increase in LFCNN. The accuracy in FCNNA was increased from 72.92% to 77.98% after channel selection. Similarly, using LFCNN, the accuracy for subject 1 was improved from 73.47% to 75.81% with channel selection. This indicates that the classifiers testing subjects one and three, highlighted in bold in the table, should be selected. Consequently, we will have two classifiers for each model, resulting in a total of eight classifiers. It is important to highlight that, when applying transfer learning using the classifier tested on subject 1 with channel selection, the optimal channels chosen for subject 1 are applied to all target subjects. The same approach is followed for subject 3.

Across the eight selected classifiers, we applied various transfer-learning strategies, including adjustments to the learning rates, division sizes, epochs, and freezing levels. In the following section, we will present the results based on these strategies.

### 5.2. Performances of the Proposed Transfer-Learning Technique Applied in the Main Models

This section analyzes the effectiveness of the proposed transfer-learning technique and provides evidence that it can enhance accuracy even when merging data from different subjects. Initially, we conducted cross-subject classification on all nine subjects without employing transfer learning, utilizing both the FCNNA and LFCNN models. This classification provides a baseline analysis, which serves as a reference for later comparisons. The results for subjects 7, 8, and 9 are presented in Table 5. The FCNNA model achieved an average accuracy of 75.79%, with individual accuracies of 72.63% for subject 7, 81.68% for subject 8, and 73.05% for subject 9. On the other hand, the LFCNN model outperformed FCNNA, with an average accuracy of 78.70%, based on individual accuracies of 76.09%, 83.55%, and 76.45% for subjects 7, 8, and 9, respectively. The table also includes the average accuracies for pairs of subjects, which can be used in future comparisons. The FCNNA model achieved two-subject averages ranging from 72.84% to 77.37%, while the LFCNN model showed higher two-subject averages, ranging from 76.27% to 80.00%.

In this section, we will demonstrate how our transfer-learning strategy can further improve these average accuracy results, particularly across different subjects.

#### 5.2.1. Transfer Learning on Three Subjects

In this study, we focus on three target subjects (subjects 7, 8, and 9), applying transfer learning to each subject’s data alternately, as described in Section 3.2.3. We used eight pre-trained classifiers and applied transfer learning across the target subjects using two division sizes—six and four—and four learning rates: LR0 = 0.0009 (same as the pre-trained model), LR1 = 0.0001, LR2 = 0.00009, and LR3 = 0.00001. Training was conducted for 30 epochs, and the freezing technique was not applied at this stage. The resulting outcomes are presented below.

**Figure 8:**
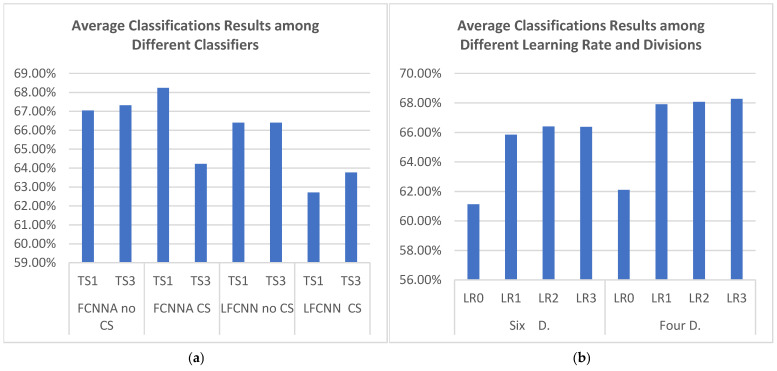
(**a**) The comparison of the average accuracy results between different testing subjects (sub1, sub3) across the four main models. (**b**) The comparison of the average accuracy results between different learning rates and data divisions.

Table 6 presents the average accuracy results obtained by applying various strategies to the eight pre-trained models. The best results, which are bolded, were achieved using the pre-trained model of testing subject 1 (TS1) with FCNNA combined with channel selection, yielding accuracies of 72.44% in the six-division setup and 74.02% in the four-division setup. Figure 8 further illustrates the impact of different learning rates and pre-trained models. As shown in Figure 8a, the FCNNA pre-trained models generally produce the best outcomes, particularly in the cases of testing subject 1 with channel selection, testing subject 1 without channel selection, and testing subject 3 without channel selection. A detailed comparison reveals that, while the model based on testing subject 1 consistently delivers the highest overall accuracy, the model based on testing subject 3 occasionally outperforms it in specific instances. Meanwhile, Figure 8b highlights that using LR0 consistently results in the lowest accuracy, regardless of whether the data are divided into six or four segments.

At this stage, we applied freezing strategies across all levels, starting with level 26, which represents a middle ground among the levels (6, 16, 26, and 44). Based on the previous results, we concentrated on the three best-performing classifiers, applying freezing at level 26 over 30 epochs and comparing the subsequent outcomes. However, we excluded LR0, as it had previously yielded the worst results. Table 7 illustrates the average accuracy results after applying transfer learning to the three target subjects alternately. We found that freezing improved accuracy by retaining certain features of the original data while incorporating features from the target data at the final stages of the model architecture. Notably, we achieved an accuracy of 78.24% in the six-division setup and 78.48% in the four-division setup using the FCNNA model without channel selection. Moreover, the efficiency of this approach was evident as freezing reduced both the time and parameters, demonstrating a significant performance enhancement. However, the best accuracy without using transfer learning, which was 78.70%, has not yet been surpassed, although we have come close. There remains a potential for further improvement by exploring other freezing levels or adjusting the number of epochs.

According to the level 26 freezing results, we selected the best pre-trained model—FCNNA from testing subject 1 without channel selection—to identify the optimal freezing level. The training was conducted over 30 epochs, using various learning rates and division sizes. As shown in Table 8, increasing the level of freezing significantly improves the results. At level 44, we achieved a higher average accuracy compared to not using transfer learning, reaching 79% in the six-division setup and 79.77% in the four-division setup. By increasing the freezing level, we ensure that the new training model captures more complex patterns from the original, suggesting that the new dataset shares similar characteristics with the dataset used for pre-training.

Given the strong accuracy achieved with level 44 freezing, we explored different numbers of epochs (30, 50, 100, 200) to assess the potential for further accuracy improvements. Table 9 illustrates how each epoch setting affects the accuracy across various learning rates and division sizes. Our findings indicate that 50 epochs yielded the highest accuracy, reaching 80.29%. However, 30 epochs consistently produced better accuracy across all conditions compared to 50 epochs. While epochs 100 and 200 also provided good accuracy, they required significantly more computations without offering any notable improvement.

Since level 44 freezing with 30 epochs yielded promising results, we applied the same conditions to the four main models on testing subject 1, exploring various learning rates and division setups to assess the potential for further accuracy improvements. However, as presented in Table 10, the FCNNA model without channel selection in testing subject 1 consistently proved to be the best-performing model. It is worth noting that the LFCNN model showed a slight improvement with freezing compared to the results in Table 6, but this improvement is not considered significant. In the experiments conducted using FCNNA, the highest average classification accuracy without channel selection was 79.77%, while with channel selection, it reached 76.90%. A closer examination of the individual subject performances reveals that, without channel selection, subject 7 achieved an accuracy of 88.28%, subject 8 attained 84.35%, and subject 9 achieved 66.67%. However, when channel selection was applied, subject 7’s accuracy dropped significantly to 79.69%, while subject 8 and subject 9’s performances remained unchanged at 84.35% and 66.67%, respectively. This suggests that the channel selection process may negatively impact some subjects more than others, particularly in the case of subject 7, indicating potential variability in the effectiveness of channel selection across different subjects.

Based on the data presented in Table 11, a comparison of efficiency versus average accuracy for subjects 7, 8, and 9 between the FCNNA and LFCNN models, both with and without transfer learning (TL), highlights several key differences. Without transfer learning, the FCNNA model requires 9 h and 30 min for cross-subject classification, achieving an average accuracy of 75.79%, with 358.20 KB of trainable parameters. In contrast, the LFCNN model completes the task in 2 h and 58 min, with a higher accuracy of 78.70% and using fewer trainable parameters 31.86 KB. When transfer learning is introduced, with four divisions (Four D.) and freezing at 44 layers (F44), the FCNNA model performs the classification in just 5 min, achieving an accuracy of 79.77% without channel selection. With channel selection, the time is reduced to 3 min, achieving an accuracy of 76.90%. In both cases, the model uses 114.53 KB of trainable parameters. The LFCNN model, with transfer learning, achieves a runtime as short as 2 min, with accuracies varying by approach, ranging from 68.85% to 72.88%. This analysis underscores the trade-off between model efficiency and performance. Although the LFCNN model is more parameter-efficient, it generally achieves higher accuracy than the FCNNA model without transfer learning. However, the effectiveness of transfer learning introduces additional considerations, balancing reduced computation time with potential impacts on accuracy. In general, the trainable parameters become the same, either with or without using channel selection, since channel value is affected at the beginning layers where these layers are frozen.

#### 5.2.2. Transfer Learning on Two Subjects and One Subject

As part of our ongoing experiments, we explore a scenario in which transfer learning is applied to the target subjects, either alternately across two subjects or individually to a single subject. This follows our earlier experiments in which transfer learning was applied across three subjects, allowing us to further investigate model adaptability and performance under different conditions. We compare the results of the four main models, each pre-trained on testing subject 1, under conditions without freezing (No F.) and with freezing applied at layer 44, and using two setups, namely six and four divisions, over 30 epochs.

**Figure 9:**
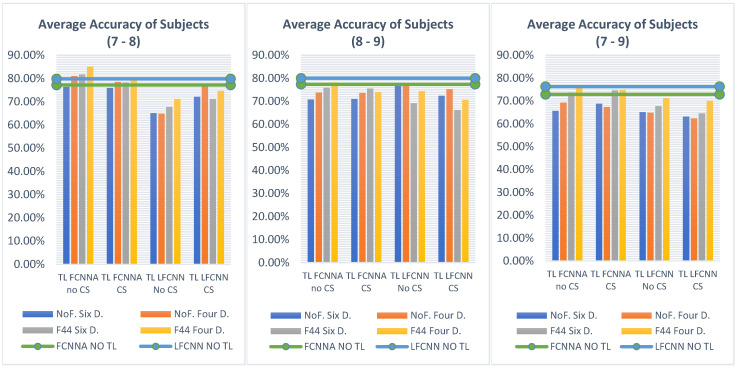
Comparison of average accuracy for transfer learning across subject pairs (7–8, 8–9, 7–9) using the pre-trained classifier obtained from testing subject 1 versus main models without transfer learning.

Starting with two-subject transfer learning, Figure 9 presents the average transfer-learning results for each two-subject combination across the selected classifiers, compared with the previously reported two-subject average accuracies without transfer learning, as shown in Table 5. Based on the averages of subjects 7 and 8, applying transfer learning with the FCNNA model improved the accuracy by approximately 6.55% without channel selection and 0.46% with channel selection. For the averages of subjects 8 and 9, the accuracy without transfer learning reached 77.37% with the FCNNA model and 80.00 percent with the LFCNN model. When applying transfer learning, the best accuracy was achieved with the FCNNA model without channel selection, improving the FCNNA accuracy by approximately 1.11% but still falling short of the LFCNN accuracy by approximately 1.77%. According to the average accuracy of subjects 7 and 9, transfer learning with the FCNNA model without channel selection achieved the highest accuracy, improving the non-transfer-learning classification accuracy by approximately 0.17 percent. Additionally, transfer learning with the FCNNA model and channel selection led to a significant improvement, increasing the non-transfer-learning accuracy of FCNNA by approximately 2.61 percent. In conclusion, the FCNNA model, both with and without channel selection, generally improved the results of non-transfer-learning classification, especially when utilizing four divisions and freezing 44 layers, which also enhanced the time and memory usage.

**Figure 10:**
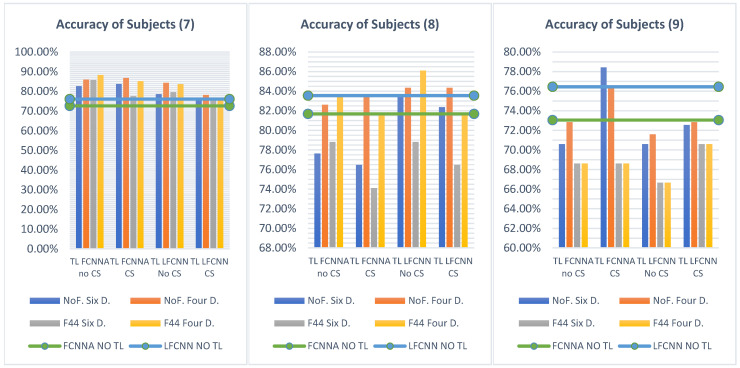
Comparison of average accuracy for single-subject transfer learning (subjects 7, 8, or 9) using the pre-trained classifier obtained from testing subject 1 versus main models without transfer learning.

Single-subject classification with transfer learning has been the standard approach in previous studies. As shown in Figure 10, transfer learning significantly improves performance, with all models in various configurations enhancing the accuracy of subject 7 by approximately 12.19 percent, increasing from 76.09% to 88.28%. For subject 8, the LFCNN model demonstrated greater improvement compared to the FCNNA model. Finally, for subject 9, improvements were only observed with the FCNNA model when using channel selection, but without freezing.

#### 5.2.3. Transfer-Learning Strategies Analysis

**Figure 11:**
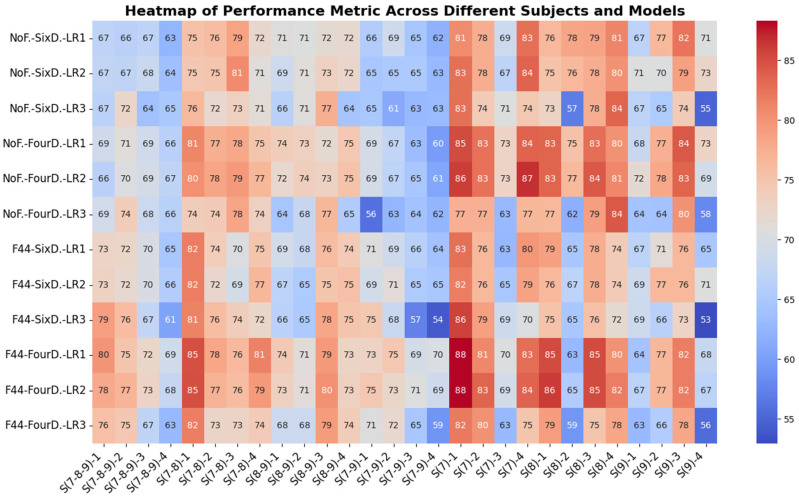
Heatmap of average accuracy for combinations of different subject groups across all main classifiers (testing subject 1). Colors range from blue (lower accuracy values) to red (higher accuracy values). The numbers on the *X*-axis represent: 1 = FCNNA without channel selection, 2 = FCNNA with channel selection, 3 = LFCNN without channel selection, and 4 = LFCNN with channel selection (all classifiers on testing subject 1).

The experiments in our study reveal an intriguing pattern in performance based on the combination of transfer-learning strategies employed (freezing levels, divisions, and learning rates) across different classifiers and subject groups. To illustrate this, Figure 11 presents a heatmap displaying the accuracy results for each subject group across all main classifiers and the various strategies used. The heatmap uses color gradients ranging from blue (indicating lower accuracy values) to red (indicating higher accuracy values) to visualize the performance metrics. From the figure, it is evident that transfer learning on a single subject generally yields better accuracy compared to multiple-subject averages. However, using two or three averaged subjects yields better results when combined with four divisions and freezing strategies. Additionally, the FCNNA model without channel selection provides the best accuracy in most scenarios. When averaging two or three subjects, FCNNA with channel selection ranks as the second-best option in terms of accuracy. On the other hand, the LFCNN model, both with and without channel selection, demonstrates strong performance in the accuracy results for individual subjects.

**Figure 12:**
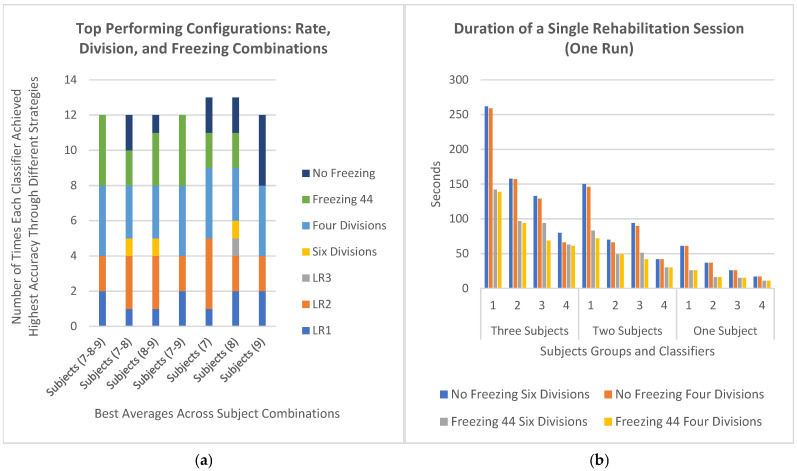
(**a**) Top performing configurations: rate, division, and freezing combinations. (**b**) duration of a single rehabilitation session in seconds. Classifier key: 1 = FCNNA no CS (TS1), 2 = FCNNA CS (TS1), 3 = LFCNN no CS (TS1), 4 = LFCNN CS (TS1).

For further clarity, we analyzed the instances where each strategy achieved the best accuracy across the 28 columns of the heatmap table, as illustrated in Figure 12a. In each subject group, the best result from each model is presented in a way that highlights the techniques used, such as freezing, division, and learning rates. The figure shows that four divisions yielded the best results, outperforming six divisions, which provided the highest accuracy in only 3 out of the 28 cases. Additionally, learning rates 2 and 1 produced higher accuracy, with learning rate 2 being the most effective. In contrast, learning rate 3 achieved the highest accuracy in only one situation. Furthermore, freezing strategies delivered the best accuracy when averaging three subjects, followed by two subjects. However, in single-subject scenarios, the non-freezing strategy performed better, which is expected since the model can more easily adapt to the specific features of that individual subject. Regarding the training time, Figure 12b illustrates the duration in seconds required to complete a single rehabilitation session, with the data divided into either four or six divisions. As previously discussed, the FCNNA model without channel selection usually produces the best results across all subject groups. However, this configuration also incurs the longest processing time, approximately 4 min for three subjects. Generally, there is a tradeoff between time and accuracy, except when applying the freezing strategy. Freezing allows for a significant reduction in processing time—by about half to one and a half minutes—while still improving accuracy, particularly in multi-subject transfer-learning scenarios.

### 5.3. Comparison of Previous Works

To demonstrate the performance of our approach, we compared it against previous work that applied cross-subject transfer learning on the BCI IV 2a dataset. In our study, we applied transfer learning exclusively to subjects 7, 8, and 9, so the comparison focuses solely on these three subjects. As previously mentioned, our study is unique in that it applies multi-subject transfer learning with multiple target subjects, but we also include cases where only one subject is used as the target. Therefore, our comparison covers all possible scenarios.

Table 12 illustrates a comparison of various approaches applied to the BCI IV 2a dataset. The paper in [9] proposed an adaptive transfer-learning approach, with the results demonstrated on the same dataset, as shown in [8]. The study in [8] also explains and presents the results of the multi-direction transfer-learning (MDTL) strategy applied to the EEGNet model (EEGNet_MDTL). Additionally, the paper in [41] conducted experiments on this dataset using two approaches: RA-MDRM, detailed in [40], and EA-CSP-LDA. The results of the MMFT and DSTL methods are presented in figures in the papers [35,39], Accordingly, the values in the following comparisons were derived directly from these figures. The papers in [37,48] represent state-of-the-art works focusing on domain adaptation techniques. While ref. [37] combined GNNs with transfer learning, ref. [48] applied cross-subject classification without transfer learning. The study in [48] incorporated a feature extraction module, including graph-related features, and integrated it with domain generalization (DG) techniques, which help extract domain-invariant features that can be effectively applied to unseen target data. For both papers, the results of cross-subject four-class classification are presented in [48]. According to the results, in our work, subject 7 consistently achieved the best performance across all scenarios, particularly in the two-subject configuration. Furthermore, our method demonstrated the highest overall average accuracy in all configurations, especially when applying two-target-subject transfer learning. This comparison highlights the effectiveness of our approach, especially for subject 7, which consistently outperforms other subjects. Moreover, our method achieves the best overall average accuracy across all scenarios.

## 6. Limitations

This study introduced multi-subject transfer learning, a promising technique that yielded interesting findings and demonstrated effective performance. However, not all possible combinations of source and target groups were tested, which limits the exploration of the impact of different groupings on the accuracy and generalizability of the framework. Future work will aim to address this limitation by testing a wider range of groupings to evaluate the framework’s performance across diverse configurations.

## 7. Conclusions

In this study, we proposed a novel multi-subject transfer-learning method to enhance online rehabilitation systems using edge computing in an IoT environment. This approach improves MI classification accuracy and enables real-time data integration of new subjects, supporting more effective rehabilitation. The architecture consists of three layers, namely cloud, edge, and sensor, each enhancing system efficiency and responsiveness. The edge layer minimizes communication latency while enabling a unified model for local predictions. This model starts as a pre-trained version in the cloud and then is retrained at the edge node using local EEG sensor data. Data are transmitted via Bluetooth to the edge gateway (Raspberry Pi) and relayed to the edge node via Wi-Fi, maintaining continuous updates.

Transfer learning for MI classification was applied using different strategies, including freezing layers, varying data divisions, and adjusting the number of epochs. These strategies were tested on two main models, FCNNA and LFCNN, both with and without channel selection. Our goal was to optimize accuracy while maintaining computational efficiency. We observed that reducing the epochs from 1000 to as low as 30 significantly improved both accuracy and efficiency, with 30 epochs achieving the best results. Freezing layers at different levels (6, 16, 26, and 44) also reduced the trainable parameters and computation time, with greater accuracy achieved as more layers were frozen, especially in the multi-subject setting.

We evaluated the proposed framework using the BCI IV 2a dataset for both multi- and single-subject transfer learning, focusing on subjects 7, 8, and 9. The highest accuracy was achieved by the FCNNA model using 30 epochs, four data divisions, and freezing 44 layers, reaching 79.77% without channel selection and 76.90% with it. In two-subject transfer learning, the accuracy improved by up to 6.55%, while single-subject transfer learning saw enhancements of about 12.19%. Notably, our approach significantly boosted the accuracy for subject 7, and the average across subjects 7, 8, and 9 surpassed previous studies.

Overall, this study demonstrates the potential of multi-subject transfer learning to enhance MI classification within an edge-computing and IoT-based rehabilitation system. The enhancements in cross-subject classification through various transfer-learning strategies, applied to both multi-subject and single-subject data, underscore the robustness of the proposed framework in adapting to diverse patient data. Future research might expand this approach to accommodate larger populations and investigate its use in a wider range of motor imagery tasks. Moreover, practical implementation and testing of the proposed system in real-world environments is an under-development task that will help to estimate through experience the real magnitude of the proposed approach. This includes addressing key challenges, such as managing network delays, ensuring efficient real-time data processing, and achieving system scalability. The findings of this study provide a foundation for creating personalized, efficient, and adaptive healthcare solutions, paving the way toward fully autonomous and practical rehabilitation systems.

## Figures and Tables

**Table 1:** Comparison of transfer learning approaches across key criteria. *X* = not applied, *✔* = applied, X* = sometimes included.

Approaches	Datasets/ # of Tasks	Target Subject Data Is Excluded from Pre-Training	The Model Is Re-Trained?	TL Is Applied to Multiple Subjects?
Zheng et al. [34] (2024)	SameDS/4classes	*X*	*X*	*X*
Zhuo et al. [38](2024)	SameDS/4classes	*X*	*X*	*X*
Li et al. [36] (2024)	SameDS/4classes	*X*	*X*	*X*
Kim et al. [10] (2023)	SameDS/4classes	X*	*X*	*X*
Li et al. [8] (2023)	SameDS/4classes	*X*	*X*	*X*
Maswanganyi et al. [35] (2023)	SameDS/4classes	*X*	*X*	*X*
Gao et al. [39] (2023)	SameDS/4classes	*X*	*X*	*X*
Miao et al. [33] (2022)	SameDS/4classes	*X*	*X*	*X*
Liang et al. [6] (2020)	SameDS/4classes	*X*	*X*	*X*
George et al. [7] (2022)	DSNEW/4classes	X*	*✔*	*X*
He et al. [41] (2020)	SameDS/4classes	*✔*	*X*	*X*
Zanini et al. [40] (2018)	SameDS/4classes	*X*	*X*	*X*
Long et al. [43] (2023)	SameDS/2classes	*✔*	*X*	*X*
Han et al. [37] (2023)	SameDS/2classes	*✔*	*✔*	*X*
Wu et al. [44] (2022)	SameDS/2classes	X*	*X*	*X*
Zhang et al. [9] (2021)	DSNEW/2classes	*X*	*X*	*X*
Our Work	SameDS/4classes	*✔*	*✔*	*✔*

**Table 2:** Modified F1 and kernel width size values for the two layers of the suggested models.

	First Layer		Second Layer	
Models	F1	KE1	F1	KE1
FCNNA Model	96	60	16	64
XFCNN Model	16	60	16	64
LFCNN Model	8	48	16	64

**Table 3:** Model efficiency comparison with accuracy results of within-subject classification.

Models	Parameters	MAC	Within-Subject Classification	
			Accuracy	Times
FCNNA Model	358.20 KB	184 M	83.78%	4:34:22
XFCNN Model	49.37 KB	50 M	82.11%	2:00
LFCNN Model	31.86 KB	35 M	81.09%	1:56:42

**Table 4:** Cross-subject classification accuracy of source subjects using FCNNA and LFCNN models, with and without channel selection, including selected channels. The abbreviations used in the Tables and Figures are listed in the acronyms section at the end of the paper.

	FCNNA No CS	FCNNA CS		LFCNN No CS	LFCNN CS	
	Acc (%)	Acc (%)	Selected Channels	Acc (%)	Acc (%)	Selected Channels
**Sub1 in testing**	**72.92**	**77.98**	**[3,8,10,11,13,15,16,18–22]**	**73.47**	**75.81**	**[3,10,13,14,17,19–22]**
Sub2 in testing	53.71	53.53	[1–3,13,17,19–22]	56.60	58.59	[2,5,6,15,17–22]
**Sub3 in testing**	**84.16**	**85.45**	**[4–11,15,16,18,20,22]**	**83.06**	**86.00**	**[2,3,5,7,8,12,14–16,18,20–22]**
Sub4 in testing	57.76	55.10	[3,5–7,12,15–19,22]	58.16	58.16	[1,4,9–11,13,19–22]
Sub5 in testing	57.43	58.74	[2,6,9,11,12,14,16,17,19,22]	56.69	53.35	[3,4,6,7,9,11,12,15,21,22]
Sub6 in testing	61.06	61.52	[1,2,5,6,11,14–17,19,21]	59.45	58.99	[2,4–6,8,10,11,13,14,17,22]
AVG	64.51	65.39		64.57	65.15	

**Table 5:** Cross-subject classification accuracy results without transfer learning for FCNNA and LFCNN models (subjects 7, 8, and 9, including overall average and two-subject averages).

	Subject 7	Subject 8	Subject 9	AVG All	AVG 78	AVG 89	AVG 79
FCNNA	72.63%	81.68%	73.05%	75.79%	77.15%	77.37%	72.84%
LFCNN	76.09%	83.55%	76.45%	78.70%	79.82%	80.00%	76.27%

**Table 6:** The average accuracy results of transfer learning across three subjects with different learning rates and different division sizes.

		FCNNA No CS		FCNNA CS		LFCNN No CS		LFCNN CS	
		TS 1	TS 3	TS 1	TS 3	TS 1	TS 3	TS 1	TS 3
Six Divisions	LR0	67.39%	61.74%	60.67%	61.27%	63.49%	61.79%	53.73%	58.97%
Six Divisions	LR1	66.89%	68.09%	66.37%	63.88%	66.94%	67.39%	62.58%	64.64%
Six Divisions	LR2	67.23%	68.72%	67.13%	62.37%	68.20%	68.70%	63.67%	65.24%
Six Divisions	LR3	66.55%	69.09%	**72.44%**	65.43%	63.83%	63.94%	64.88%	64.88%
Four Divisions	LR0	64.10%	63.83%	64.05%	61.50%	62.21%	62.61%	57.09%	61.45%
Four Divisions	LR1	68.77%	68.67%	70.96%	66.58%	68.82%	68.31%	66.39%	64.79%
Four Divisions	LR2	66.39%	69.24%	70.28%	68.35%	69.37%	69.56%	67.09%	64.27%
Four Divisions	LR3	69.08%	69.19%	**74.02%**	64.45%	68.36%	68.95%	66.25%	65.89%

The bolded values indicate the best results.

**Table 7:** Transfer learning average accuracy using Freezing at level 26.

			FCNNA No CS		FCNNA CS
			TS 1	TS 3	TS 1
Freezing 26	Six Divisions	LR1	73.83%	70.76%	71.05%
	Six Divisions	LR2	70.38%	70.32%	70.76%
	Six Divisions	LR3	68.28%	66.53%	75.34%
	Four Divisions	LR1	**78.24%**	76.45%	74.33%
	Four Divisions	LR2	**78.48%**	75.59%	71.71%
	Four Divisions	LR3	75.51%	71.24%	73.78%

The bolded values indicate the best results.

**Table 8:** Transfer learning average accuracy across various freezing levels using FCNNA of testing subject 1 model without channel selection.

			Freezing Levels			
			6	16	26	44
FCNNA no CS—TS 1	Six Divisions	LR1	70.53%	68.80%	73.83%	72.88%
	Six Divisions	LR2	66.55%	68.02%	70.38%	73.22%
	Six Divisions	LR3	65.69%	62.34%	68.28%	**79.00%**
	Four Divisions	LR1	71.52%	72.46%	78.24%	**79.77%**
	Four Divisions	LR2	71.81%	71.76%	**78.48%**	78.06%
	Four Divisions	LR3	69.43%	65.61%	75.51%	75.60%

The bolded values indicate the best results.

**Table 9:** Transfer learning average accuracy of level 44 freezing across different epochs.

			Freezing 44			
Epochs			30	50	100	200
FCNNA no CS—TS 1	Six Divisions	LR1	72.88%	72.96%	74.35%	75.68%
	Six Divisions	LR2	73.22%	72.28%	76.44%	75.71%
	Six Divisions	LR3	**79.00%**	69.41%	72.83%	72.15%
	Four Divisions	LR1	**79.77%**	79.24%	78.11%	77.78%
	Four Divisions	LR2	78.06%	**80.29%**	79.70%	78.85%
	Four Divisions	LR3	75.60%	74.51%	77.27%	78.25%

The bolded values indicate the best results.

**Table 10:** Transfer learning average accuracy in level 44 freezing across various classifiers.

			FCNNA No CS	FCNNA CS	LFCNN No CS	LFCNN CS
			TS 1	TS 1	TS 1	TS 1
Freezing 44	Six Divisions	LR1	72.88%	72.41%	69.93%	64.64%
	Six Divisions	LR2	73.22%	72.10%	69.98%	65.51%
	Six Divisions	LR3	**79.00%**	76.05%	67.49%	60.51%
	Four Divisions	LR1	**79.77%**	74.95%	72.45%	68.85%
	Four Divisions	LR2	78.06%	76.90%	72.88%	67.85%
	Four Divisions	LR3	75.60%	74.74%	67.04%	62.91%

The bolded values indicate the best results.

**Table 11:** The efficiency comparisons between the main pre-trained classifiers and the main chosen models based on cross-subject classification.

Models	Trainable Parameters	Cross-Subject Classification	
		AVG Accuracy	Times
FCNNA Model No TL	358.20 KB	75.79%	9 h, 30 min
LFCNN Model No TL	31.86 KB	78.70%	2 h, 58 min
FCNNA No CS using TL (TS1, F44, Four D.)	114.53 KB	79.77%	5 min
FCNNA CS using TL (TS1, F44, Four D.)	114.53 KB	76.90%	3 min
LFCNN No CS using TL (TS1, F44, Four D.)	18.28 KB	72.88%	3 min
LFCNN CS using TL (TS1, F44, Four D.)	18.28 KB	68.85%	2 min

**Table 12:** A comparison of transfer-learning accuracies for previous approaches on the BCI IV 2a dataset. * Approximated value based on figure.

	Adaptive TL [8]	RA-MDRM [41]	EA-CSP-LDA [41]	EEGNet-MDTL [8]	MMFT [35]	Li et al. [36]	TL-GNN [48]	STG-DAN [48]	DSTL [39]	Our Work (Three-Target Subjects)	Our Work (Two-Target Subjects)	Our Work (One-Target Subjects)
Sub 7	76.39	61.81	68.75	80.56	68.5 *	53.91	70.52	73.92	70 *	89.84	91.41	88.28
Sub 8	74.31	86.81	89.58	75.69	76 *	69.89	68.33	68.22	91.6 *	84.35	86.09	86.09
Sub 9	64.58	82.64	72.92	79.17	36 *	71.56	65.70	66.09	75.7 *	66.67	70.37	72.84
AVG	71.76	77.09	77.08	78.47	60.17 *	65.12	68.18	69.41	79.1 *	80.29	82.62	80.81

## Data Availability

The BCI-IV-2a dataset can be downloaded from the following link: https://www.bbci.de/competition/iv/#datasets, accessed on 10 March 2023.

## Acronyms

**Symbol**	**Full Name**
Acc	Accuracy
CS	Channel Selection
F44	Freezing 44
Four D.	Four Divisions
LR	Learning Rate
NoF.	No Freezing
S(7-8-9)	Subjects (7-8-9)
Six D.	Six Divisions
TL	Transfer Learning
TS	Testing Subject

## Appendix A

The proposed system involves IoT devices and servers. In this section, we detail the data flow and evaluate the suitability of the Raspberry Pi and Bluetooth for handling the expected data volume. EEG signals are represented by a large volume of data that requires significant memory and processing power, which the cloud and edge node servers can manage efficiently. Therefore, our focus is on ensuring that the client side, such as Raspberry Pi devices with limited processing power, energy, and Bluetooth connectivity, can operate effectively. On the client side, EEG signals are transmitted via Bluetooth to an edge gateway, which includes a Raspberry Pi, and then sent to the edge node. We assess the amount of raw data transmitted online and its sufficiency. All of the 22 channels will be sent to the Raspberry Pi via Bluetooth, and later, some channels will be dropped according to the classification approach used. Consequently, we will eventually have either 22, 13, 12, or 9 channels saved in the memory of the edge gateway.

We consider scenarios involving saving data of different channel configurations, namely 22 channels, 13 channels, 12 channels, and 9 channels, each sampled at 250 Hz and lasting 7 s per trial, with a maximum of 140 trials per rehabilitation session. To calculate the memory consumption for the Raspberry Pi, we first need to determine the total number of samples and bytes per trial. Since each trial has a duration of 7 s and is sampled at a rate of 250 Hz, this results in a total of 1750 samples per channel per trial. Upon inspection of the data file, we found that the data are recorded with a precision of 64 bits (8 bytes) per sample. This setup leads to varying memory requirements depending on the number of channels used. For the 22-channel configuration, the total number of samples per session is as shown in Equation (A1):

Total number of samples: 1750 samples/trial × 22 channels × 140 trials = 5,390,000 samples (A1)

Based on the total number of sample results, the memory occupancy can be calculated as demonstrated in Equation (A2):

Total bytes: 5,390,000 samples × 8 bytes/sample = 43,120,000 bytes ≈ 41.12 MB (A2)

So as a result, the data of one rehabilitation session, while dealing with all 22 channels, will be around 41.12 MB. By applying the same process to calculate the memory occupancy for 13, 12, and 9 channels, we obtain approximately 24.29 MB, 22.43 MB, and 16.82 MB, respectively. Given the memory requirements for the largest configuration (22 channels, approximately 41.12 MB), the Raspberry Pi 4B model—available with 2GB, 4GB, and 8GB of RAM—provides sufficient memory capacity to handle the data volume for each of the channel configurations mentioned.

For Bluetooth transmission, the data from 22 channels will be sent per trial to be eventually gathered and processed at the edge gateway. By transmitting each trial separately, the transmission times are kept within approximately one second, ensuring efficient data transmission with minimal delay, as detailed below. For the 22-channel configuration, each trial includes 38,500 samples, resulting in 308,000 bytes per trial, approximately 300.8 KB, as shown in the following Equations (A3) and (A4):

Total number of samples per trial: 1750 samples × 22 channels = 38,500 samples (A3)

Total bytes per Trial: 38,500 samples × 8 bytes/sample = 308,000 bytes ≈ 300.8 KB (A4)

Bluetooth 5.0 supports a maximum data rate of up to 2 Mbps (250,000 bytes per second) [49]. By transmitting each trial separately, we can effectively manage the data flow and minimize delays. For the 22-channel configuration, the data per trial is 308,000 bytes, resulting in a transmission time of approximately 1.232 s, as shown in the following calculation:

(A5) $$\mathrm{Transmission}\;\mathrm{Time}:\;308,000\;bytes÷250,000\frac{bytes}{s}\approx 1.232\;s$$

When the edge gateway receives each trial, certain channels may be dropped based on the classification technique employed. After gathering all the trials, the information is transmitted to the edge node. As demonstrated in previous studies [50,51,52], the Raspberry Pi 4B has been effectively used for the preprocessing, feature extraction, and classification of EEG signals. Consequently, it is feasible to utilize Raspberry Pi 4B for transmitting information and performing partial preprocessing, such as dropping specific channels.
